# Complete Genome Sequence for the *Fusarium* Head Blight Antagonist *Bacillus amyloliquefaciens* Strain TrigoCor 1448

**DOI:** 10.1128/genomeA.00219-14

**Published:** 2014-03-27

**Authors:** Beth A. Nelson, Preethi Ramaiya, Alfredo Lopez de Leon, Ravi Kumar, Austin Crinklaw, Eliana Jolkovsky, Julia M. Crane, Gary C. Bergstrom, Michael W. Rey

**Affiliations:** a Novozymes, Inc., Davis, California, USA; b Davis High School, Davis, California, USA; c Department of Plant Pathology and Plant-Microbe Biology, Cornell University, Ithaca, New York, USA

## Abstract

We present the complete genome sequence for *Bacillus amyloliquefaciens* TrigoCor 1448 (ATCC 202152), a bacterial biological control agent for *Fusarium* head blight in wheat. We compare it to its closest relative, *B. amyloliquefaciens* strain AS43.3.

## GENOME ANNOUNCEMENT

In North America, the filamentous fungus *Fusarium graminearum* is the primary causal agent of *Fusarium* head blight (FHB), a major disease of wheat and barley. FHB infections can result in economically devastating reductions to grain yield and quality: during the period of 1993 to 2001, a series of FHB epidemics in the United States resulted in losses approximating $2.5 billion (1). Traditional management methods utilize fungicides, and resistant cultivars provide inconsistent and often limited control of FHB (2, 3); therefore, alternative options are increasingly being explored. In particular, researchers have evaluated the potential of managing FHB with biological control agents (4). *Bacillus amyloliquefaciens* strain TrigoCor 1448 (TrigoCor) (5) is a promising FHB biological control agent isolated from the rhizosphere of a wheat plant. Like other *Bacillus* strains being developed for FHB biological control (6, 7), TrigoCor inhibits *F. graminearum* growth *in vitro* (8, 9) and reduces FHB symptoms on wheat spikes (10, 11). The complete genome sequence for TrigoCor would provide deeper insights on how this and other *Bacillus* biological control agents antagonize FHB.

Using the CLC Workbench (CLC bio, Arhaus, Denmark), the TrigoCor genome sequence was assembled from 7,544,540 paired-end Illumina reads, each 150 bp in length. Gaps were closed using GapFiller (12) and PCR with subsequent Sanger sequencing. The assembly of all reads resulted in a single contig of 3,957,904 bp, with an average genome coverage depth of 232×. The completed genome was machine annotated using RAST 4.0 (13).

A phylogenomic comparison of the TrigoCor genome to the 19 *B. amyloliquefaciens* genomes currently available in the NCBI database was done using progressiveMauve (14). This comparison indicates that TrigoCor belongs to *B. amyloliquefaciens* subsp. *plantarum*, a subspecies that encompasses plant-associated strains (15). TrigoCor is most closely related to another potential FHB biocontrol agent, *B. amyloliquefaciens* strain AS43.3 (NRRL B-30211) (16), which was isolated from the surface of a wheat spike (6). A comparison of the TrigoCor genome sequence to that of AS43.3 shows that although the two genomes retain a high degree of synteny, they are distinct isolates. Disruptions in the synteny between the TrigoCor and AS43.3 genomes appear to be the result of loss or integration of prophages, as identified by PHAST (17), notably an insertion of a 26-kb prophage at base pair position 161000 in TrigoCor and the insertion of a 45.8-kb prophage at position 1170310 in AS43.3. In addition, TrigoCor harbors nine copies of an IS*Bsu1*-like insertion sequence, whereas AS43.3 has only three.

For both TrigoCor and AS43.3, the cyclic lipopeptides iturin and fengycin have been identified as playing a major role in the antibiosis of *F. graminearum* (9, 18, 19). Secondary metabolite clusters were identified in the genomes of these two strains using antiSMASH 2.0 (20). With the exception of the absence of the *nrs* operon in AS43.3, the two strains have similar complements of predicted secondary metabolite gene clusters in their genomes.

### Nucleotide sequence accession number.

The genome sequence of *B. amyloliquefaciens* TrigoCor 1448 has been deposited at NCBI under the accession no. CP007244.
